# Giant intracardiac blood cyst: assessing the relationship between its formation and previous cardiac surgery

**DOI:** 10.1007/s12471-015-0707-4

**Published:** 2015-06-05

**Authors:** J. Halim, F.R.N. van Schaagen, R.K. Riezebos, S. Lalezari

**Affiliations:** Onze Lieve Vrouwe Gasthuis, PO Box 95500, 1090 HM Amsterdam, The Netherlands

**Keywords:** Cardiology, Intracardiac tumours, Blood cyst, Cardiovascular surgery, Transoesophageal echocardiogram

## Abstract

This case report discusses a 23-year-old male patient who presented with shortness of breath during exercise. Echocardiography revealed an intracardiac mass located on the mitral valve. His medical history included surgical closure of an atrial septal defect type 2 at the age of 2 years. After being discussed within a multidisciplinary heart team, the intracardiac mass was surgically removed. Histopathological examination revealed a blood cyst. This case report emphasises that the presence of an acquired intracardiac cyst is a rare entity and that a pathophysiological association between the formation of a blood cyst and previous cardiac surgery has not been proven yet.

**Electronic supplementary material:**

The online version of this article (doi:10.1007/s12471-015-0707-4) contains supplementary material, which is available to authorized users.

## Introduction

In the majority of cases, intracardiac masses are tumours; 90 % of all tumours in the heart are benign. Of these tumours, myxoma is the most common (70 %). Less frequently, blood cysts are seen. A congenital origin has been described in most of these cases [1]. The exact mechanism of the formation of an acquired blood cyst is still unknown. In this article, we describe a case of a 23-year-old man in whom the formation of an acquired blood cyst was preceded by cardiac surgery. Based on current hypotheses, this pathophysiological association could be supported; however, evidence from empirical research is lacking [2].

A 23-year-old male patient presented to the outpatient clinic with shortness of breath during exercise. At the age of 2 years, he underwent surgical closure of an atrial septal defect type 2. At the age of 18 years, a mitral valve prolapse of the anterior mitral valve leaflet was diagnosed, with trivial mitral regurgitation. His history included coloboma of the iris and a surgical correction of phimosis. Physical examination was unremarkable. The electrocardiogram, laboratory testing and chest X-ray showed no abnormalities.

**Fig. 1:**
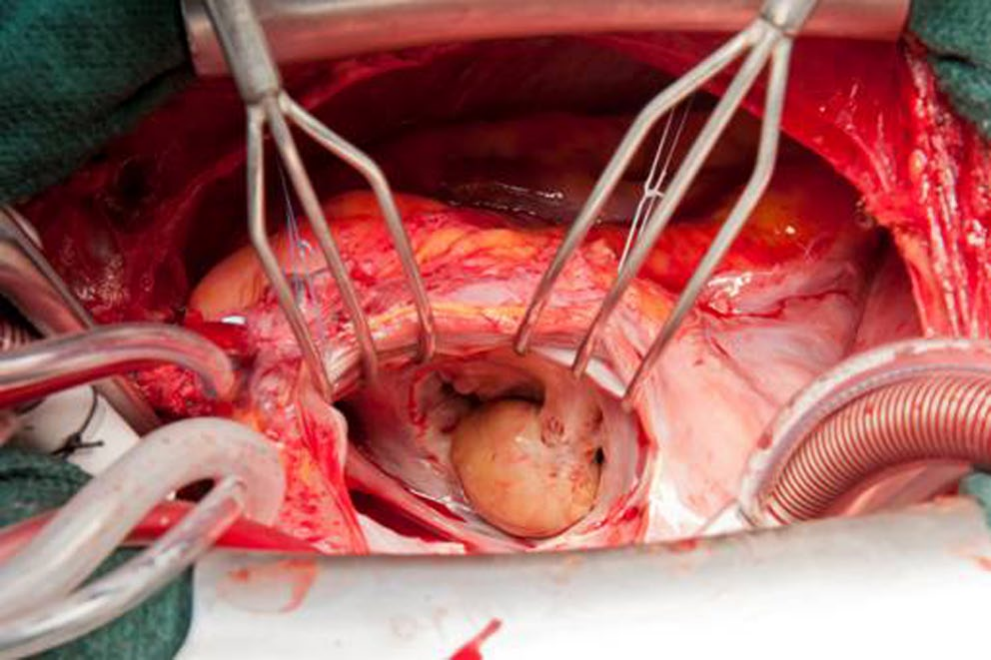
Perioperative view of the intracardiac blood cyst; the blood cyst attached on the mitral valve is clearly visible perioperatively

Transthoracic and transoesophageal echocardiography exhibited an intracardiac mass attached to the anterior leaflet of the mitral valve, a moderate mitral regurgitation with an eccentric jet as well as normal left ventricular function (Fig. 1, Movie 1). This finding made it highly probable that the intracardiac mass was a myxoma. Computed tomography angiography showed normal coronary artery anatomy. Our heart team recommended surgery because of the symptomatic nature of the intracardiac mass and the high probability of it being a myxoma. Furthermore, magnetic resonance imaging (MRI) was not performed, as the results of an MRI would not have had a significant impact on the decision to perform an operation.

## Treatment

**Fig. 2:**
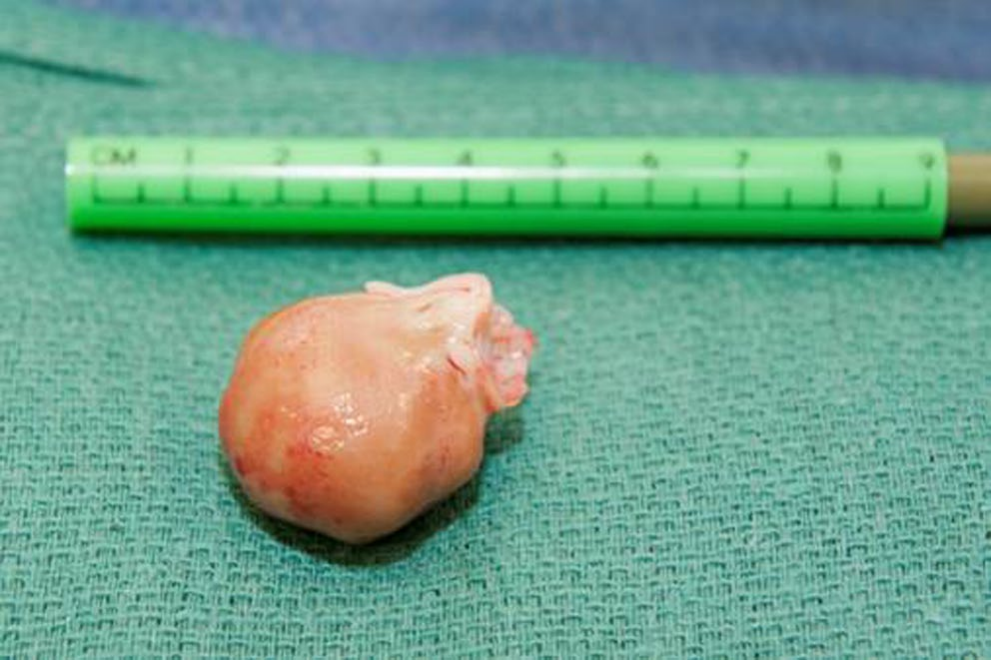
Intracardiac blood cyst: blood cyst after resection

Surgery was performed by re-do median sternotomy. After aortic and bi-caval cannulation, cardiopulmonary bypass was established. Myocardial protection was achieved by antegrade cold blood cardioplegia during aortic cross clamping. A trans-septal approach was used to expose the mitral valve and the mass. A yellow, smooth and spherical mass with a diameter of 2.0 cm was found attached to the anterior mitral leaflet adjacent to the posteromedial commissure (Fig. 2). The mass was excised, and subsequently, the mitral valve was repaired by performing annular plication at the posteromedial commissure and a sliding plasty of P3 followed by a ring annuloplasty with a 40-mm Carpentier–Edwards Physio ring.

## Results

Postoperative transoesophageal echocardiography showed no residual mitral regurgitation. The postoperative course was uneventful. On histopathological examination, the walls of the mass consisted of connective fibrous tissue. The cyst contained old blood and fibrin. No evidence of neoplastic cells was seen. The patient remained asymptomatic at 6-month follow-up.

## Discussion

A myxoma is most frequently seen as the cause of an intracardiac mass [3]. Other possible causes would be an intracardiac cyst, a fibroelastoma, cardiac metastasis or vegetation on the mitral valve in the context of endocarditis [4]. The diagnosis is made by means of echocardiography; however, this cannot reveal the exact cause. MRI has proven to be a superior tool for the assessment of intracardiac masses and its ability to differentiate between the possible causes [5]. However, histopathological examination is still the golden standard.

Intracardiac blood cysts are mostly congenital and usually regress in the first year of life [6]. The finding of an acquired blood cyst in a patient with a previous history of cardiac surgery has been described before [7]. However, there is no known causal association between its development and previous cardiac surgery. A few hypotheses have been suggested regarding the formation of these cysts. One of these hypotheses proposed that inflammation, vagal stimulation, anoxia or haemorrhagic events can result in intracardiac blood cyst formation due to haematoma formation [8]. This hypothesis could explain that intracardiac surgery provides an additional risk of formation of a blood cyst. However, due its rarity, the exact pathophysiological mechanism still needs to be elucidated.

Blood cysts are asymptomatic in most cases. The symptoms merely depend on mechanical interference with the intracardiac haemodynamic and valvular function. The most common symptoms are shortness of breath, fatigue and chest pain. Symptoms resulting from embolisation are rare [9]. In this patient, the indication for surgery was clear because of the symptoms. In asymptomatic patients, decision-making is more difficult because no specific guideline is available. A multidisciplinary heart team approach is therefore essential.

## Conclusion

Intracardiac blood cysts should be included in the differential diagnosis of intracardiac masses. MRI as a non-invasive imaging technique is beneficial to help differentiate between the possible causes of intracardiac masses. Hypotheses make it likely that there is a relationship between the formation of intracardiac cysts and a previous cardiac surgery. There are no specific guidelines for treatment of intracardiac cysts. A multidisciplinary heart team is therefore necessary to evaluate each case separately.

### Funding

None.

### Conflicts of interest

None declared.

## Electronic supplementary material

(AVI 14,652 kb)

## Movie legends

**Movie 1** Intracardiac blood cyst on the mitral valve. Parasternal long axis where a blood cyst attached on the mitral valve is visible.
